# Supplementary material for: Gradient tungsten-doped Bi3TiNbO9 ferroelectric photocatalysts with additional built-in electric field for efficient overall water splitting
Source: Nat Commun. 2023 Dec 1;14:7948. doi: 10.1038/s41467-023-43837-4 (PMC10692145; doi:10.1038/s41467-023-43837-4)
Supplement: Supplementary file 1 — Supplementary Information [file 41467_2023_43837_MOESM1_ESM.pdf]

Supplementary Information

Gradient tungsten-doped Bi_3TiNbO_9 ferroelectric photocatalysts with additional built-in electric field for efficient overall water splitting

Jie Huang[1,2#], Yuyang Kang[1#], Jianan Liu[1,2#], Tingting Yao[1], Jianhang Qiu[1], Peipei Du[3], Biaohong Huang[1], Weijin Hu[1,2], Yan Liang[1], Tengfeng Xie[4], Chunlin Chen[1], Li-Chang Yin[1], Lianzhou Wang[5], Hui-Ming Cheng[1,6], Gang Liu*[1,2]

[1] Shenyang National Laboratory for Materials Science, Institute of Metal Research, Chinese Academy of Sciences, 72 Wenhua Road, Shenyang 110016, China.

[2] School of Materials Science and Engineering, University of Science and Technology of China, 72 Wenhua Road, Shenyang 110016, China.

[3] National Laboratory of Solid State Microstructures, College of Engineering and Applied Sciences, Nanjing University, Nanjing, Jiangsu 210093, China.

[4] College of Chemistry, Jilin University, Changchun, 130012, China.

[5] Nanomaterials Centre, School of Chemical Engineering and Australian Institute for Bioengineering and Nanotechnology, The University of Queensland, QLD 4072, Australia.

[6] Institute of Technology for Carbon Neutrality, Shenzhen Institute of Advanced Technology, Chinese Academy of Sciences, Shenzhen 518055, China

[#]These authors contributed to this work equally.

Correspondence: gangliu@imr.ac.cn

Supplementary Tables

Supplementary Table 1. Photon Flux in different illuminated areas at full spectrum and monochromatic light (365 nm)

Area	Photon Flux (μmol s^{-1} m^{-2})	
	Full spectrum	365 nm
1	38003	471.09
2	8782.4	94.438
3	8749.7	103.88
4	7367.7	95.219
5	8618.3	92.931

The detailed apparent quantum yield (AQY) calculation process via photocatalytic hydrogen evolution is based on equation:

$$\mathrm{AQY} = \frac{\text{Number of reacted electrons}}{\text{Number of incident photons}} \times 100\%$$

$$= \frac{\text{Number of evolved } \mathrm{H_2} \text{ molecules} \times 2}{\text{Photon Flux} \times S \times t} \times 100\%$$

$$= \frac{\text{Number of evolved } \mathrm{H_2} \text{ molecules} \times 2}{(P_1 \times \frac{1}{3} + \frac{P_2 + P_3 + P_4 + P_5}{4} \times \frac{2}{3}) \times S \times t} \times 100\%$$

Where, S is the irradiation area (m^2), t is the photoreaction time (s), P_1, P_2, P_3, P_4 and P_5 represent the Photon Flux of the five points in Supplementary Fig. 17.

Supplementary Table 2. Performance comparison of W-doped Bi_3TiNbO_9 with previously reported ferroelectric photocatalysts claimed.

Photocatalysts	H_2 evolution rate (μmol h^{-1})	O_2 evolution rate (μmol h^{-1})	AQY (%)	Ref
Bi_3TiNbO_9-W	106.71	47.94	0.57% (365 nm)	This work
Bi_3TiNbO_9	21.78	9.94	0.26% (365 nm)	1
$PbTiO_3$	3.29	1.74	0.071 (365 nm)	2
$BaTiO_3$/Au	~0.89 μmol cm^{-2}	~0.47μmol cm^{-2}	—	3
Bi_3TiNbO_9	17.13	13.76	—	4*
$Bi_3Ti_{0.8}Cr_{0.1}Nb_{1.1}O_9$	41.11	—	0.52% (⩾ 250 nm)	5*
Bi_3TiNbO_9/RGO	2.4	—	—	6*

* represents the photocatalytic water splitting half reaction

Supplementary Figures

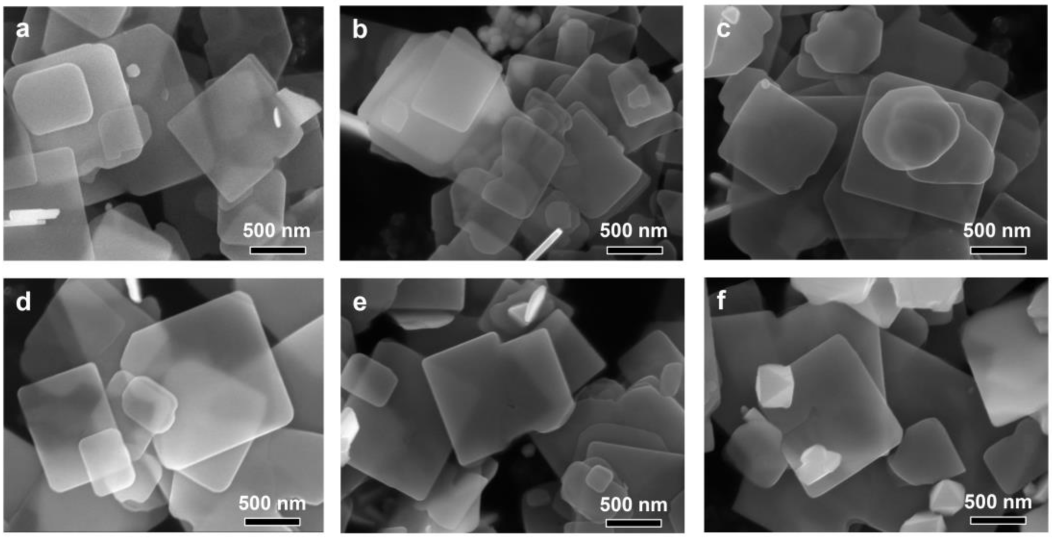

Supplementary Fig. 1 Scanning electron microscope images of Bi_3TiNbO_9-*x* W: **a** *x*=0, **b** *x*=1%, **c** *x*=3%, **d** *x*=5%, **e** *x*=7%, **f** *x*=12%.

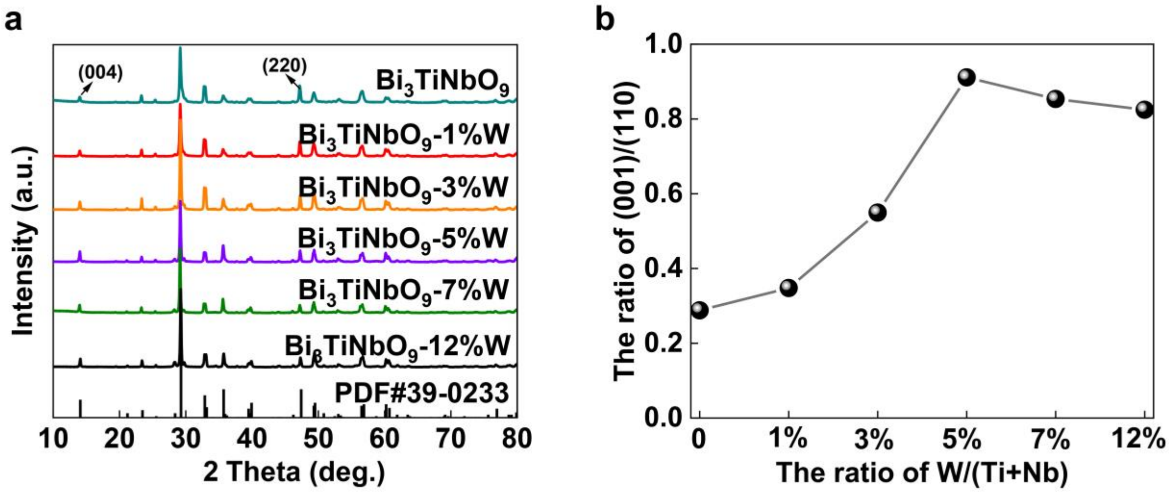

Supplementary Fig. 2 a X-ray diffraction patterns of Bi_3TiNbO_9-*x* W (*x*= 0, 1%, 3%, 5%, 7%, 12%). **b** The variation of ratio of (001)/(110) calculated from the diffraction peaks with the increase of W dopant.

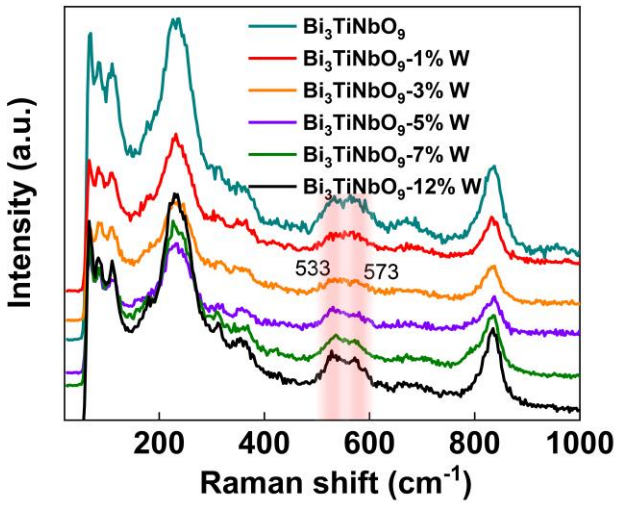

Supplementary Fig. 3 The Raman spectra of Bi_3TiNbO_9-*x* W (*x*= 0, 1%, 3%, 5%, 7%, 12%, Excitation wavelength of 532 nm).

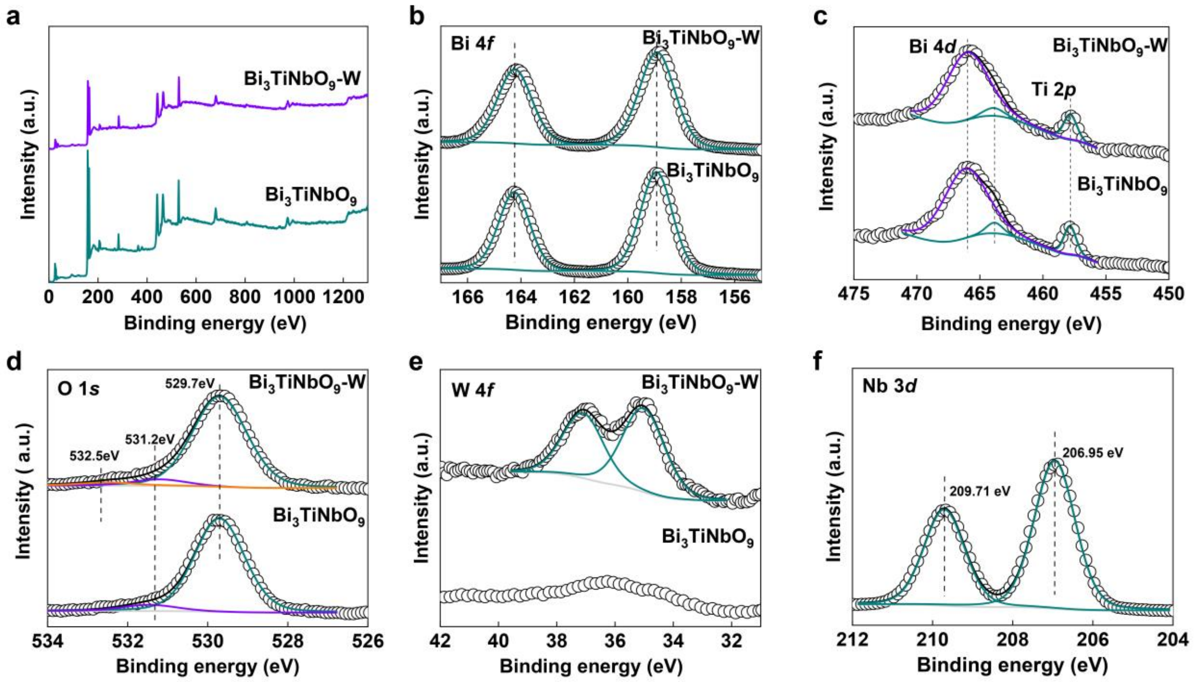

Supplementary Fig. 4 X-ray photoelectron spectroscopy results. **a** Survey spectra and **b-e** Binding energy comparison of different elements (Bi, Ti, O and W) in Bi_3TiNbO_9 and Bi_3TiNbO_9-W, **f** Nb 3*d* binding energy of commercial Nb_2O_5.

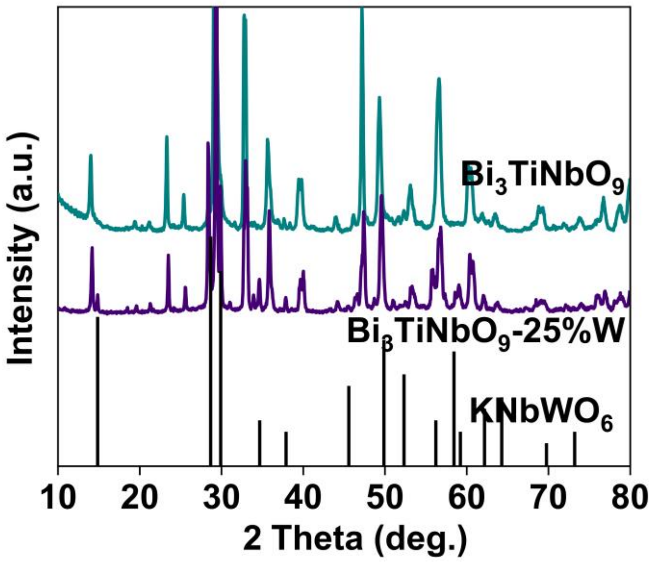

Supplementary Fig. 5 X-ray diffraction patterns of Bi_3TiNbO_9 and Bi_3TiNbO_9-25% W. The doping amount of W is further increased to ensure that the excess W reacts adequately with the substituted Nb.

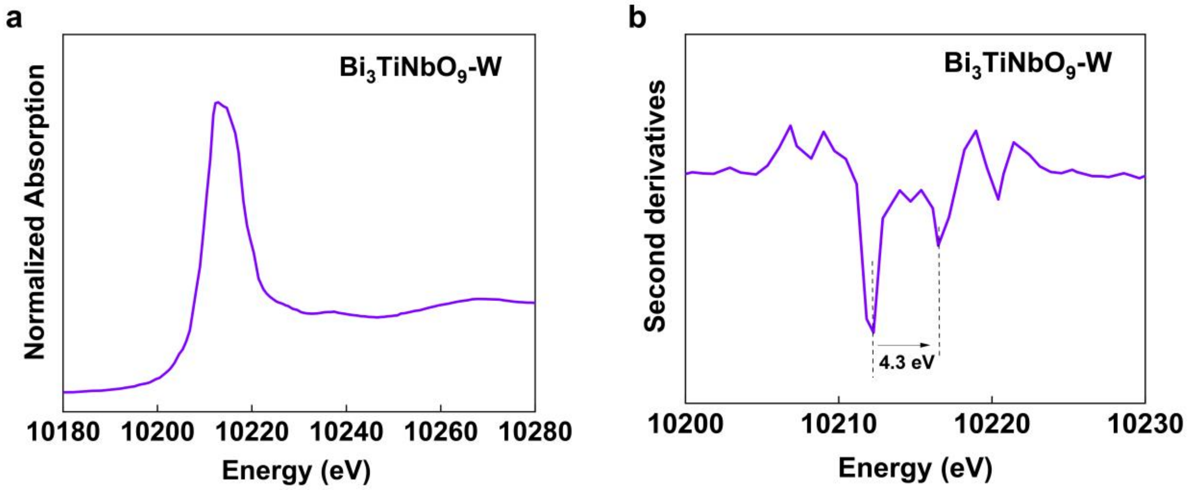

Supplementary Fig. 6 **a** W L_3-edge XANES spectra of Bi_3TiNbO_9-W. **b** Second derivatives of W L_3-edge XANES spectra.

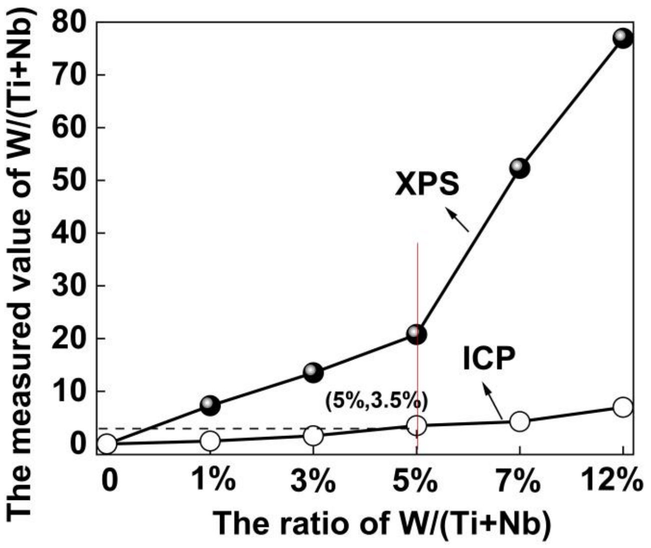

Supplementary Fig. 7 Comparison of the molar ratios of W to Ti and Nb in different doped samples measured by X-ray photoelectron spectroscopy measurements and ICP-OES results of Bi_3TiNbO_9-*x* W (*x*= 0, 1%, 3%, 5%, 7%, 12%).

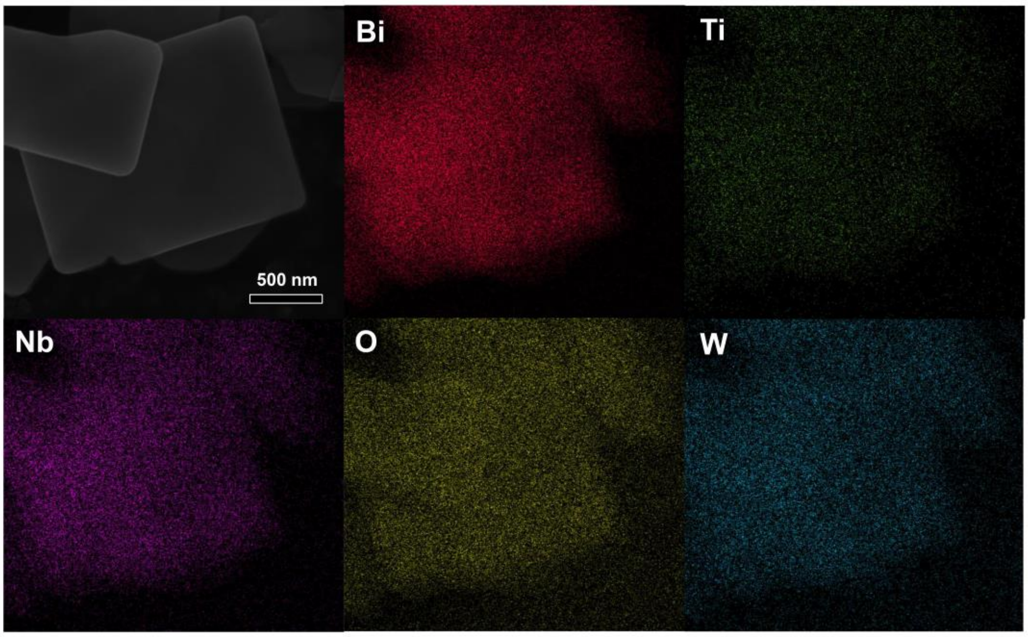

Supplementary Fig. 8 Scanning Electron Microscopy/Energy Dispersive X-Ray Spectroscopy (SEM/EDS) images of Bi_3TiNbO_9-W.

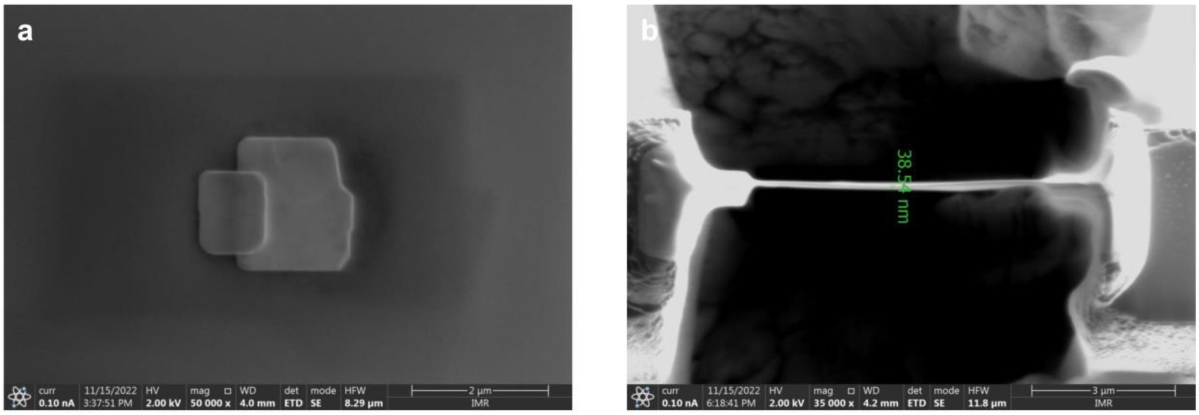

Supplementary Fig. 9 The focused ion beam (FIB) cutting process. **a** SEM image of the two stacked Bi_3TiNbO_9-W nanosheets. **b** SEM image of the nanosheets after cutting along the *c*-axis.

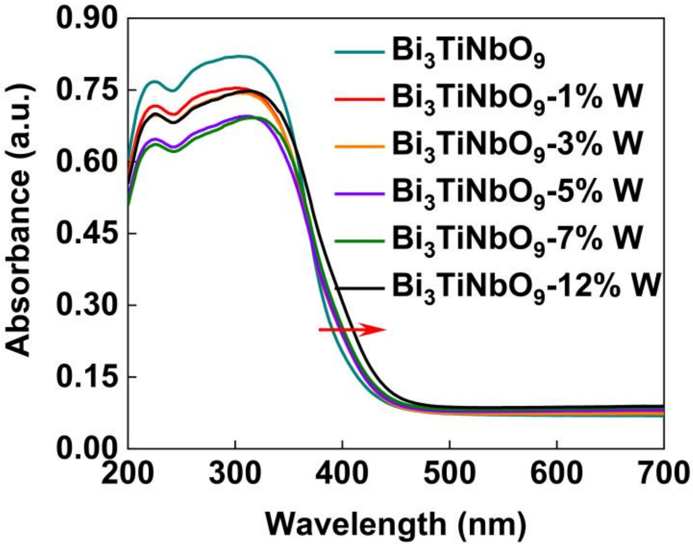

Supplementary Fig. 10 UV–visible absorption spectra of Bi_3TiNbO_9-x W (x= 0, 1%, 3%, 5%, 7%, 12%).

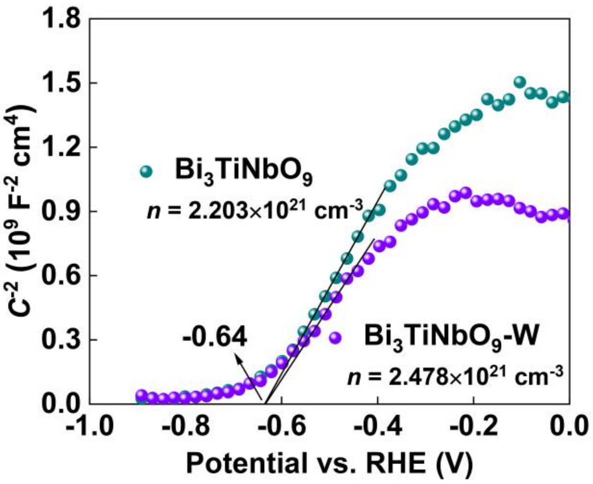

Supplementary Fig. 11 Mott-Schottky curves of Bi_3TiNbO_9 and Bi_3TiNbO_9-W.

The Mott-Schottky curves exhibit a positive slope, indicating that Bi_3TiNbO_9 and Bi_3TiNbO_9-W are n-type semiconductor. According to Fig. 4f, the dielectric constants of Bi_3TiNbO_9 and Bi_3TiNbO_9-W at 1000 Hz are 16.76 and 21.42, and the corresponding carrier concentrations are calculated to be 2.203×10^{21} cm^{-3} and 2.478×10^{21} cm^{-3}, respectively. The W dopant serve as shallow donors can effectively increase the carrier density in Bi_3TiNbO_9, thereby raising the Fermi level[7].

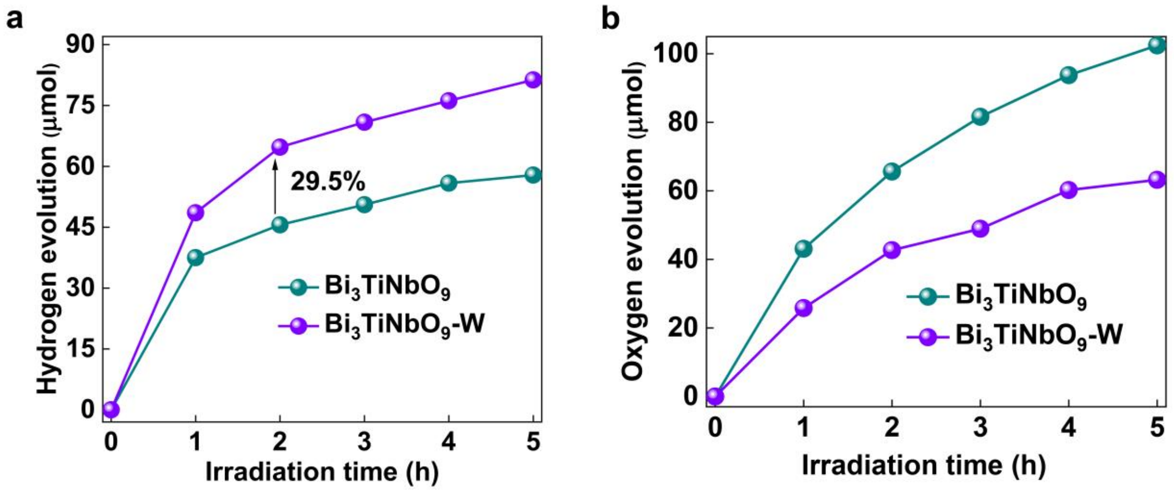

Supplementary Fig. 12 Comparison of half reactions in photocatalytic water splitting: **a** Hydrogen evolution (reaction conditions: 50 mg photocatalyst, 1 wt% Pt cocatalyst, 10 mL methanol + 90 mL H_2O). **b** Oxygen evolution (reaction conditions: 50 mg photocatalyst, 850 mg $AgNO_3$, 100 mL H_2O).

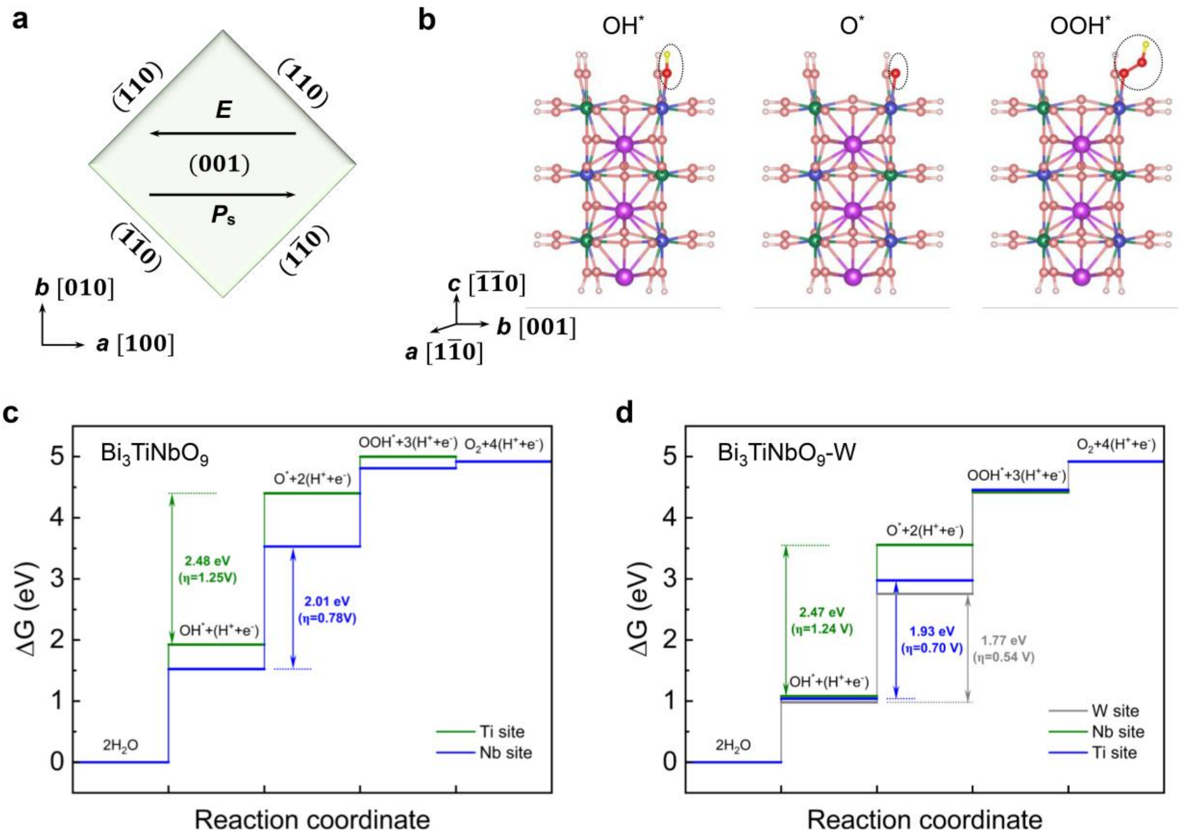

Supplementary Fig. 13 **a** Schematic diagram of the exposed $\{110\}$ facets of Bi_3TiNbO_9. The arrows indicate the spontaneous polarization (P_s) direction and the depolarization field (E) direction. **b** The atomic structures of simplified model for the $(\bar{1}\bar{1}0)$ facet of Bi_3TiNbO_9. From left to right, adsorption configurations for three intermediates (OH^*, O^*, OOH^*) of the four-electron oxygen evolution reaction on the $(\bar{1}\bar{1}0)$ facet of Bi_3TiNbO_9 are presented. The purple, green, blue, light red, and white balls represent Bi, Ti, Nb, O, and H atoms, respectively. The O and H atoms of intermediates highlighted in the dotted ellipses are denoted by bright red and yellow balls, respectively. The DFT calculation of the reaction coordinate of the four-electron oxygen evolution reaction at different active sites (Ti, Nb, W) on the facet $(\bar{1}\bar{1}0)$: **c** Bi_3TiNbO_9, **d** Bi_3TiNbO_9-W.

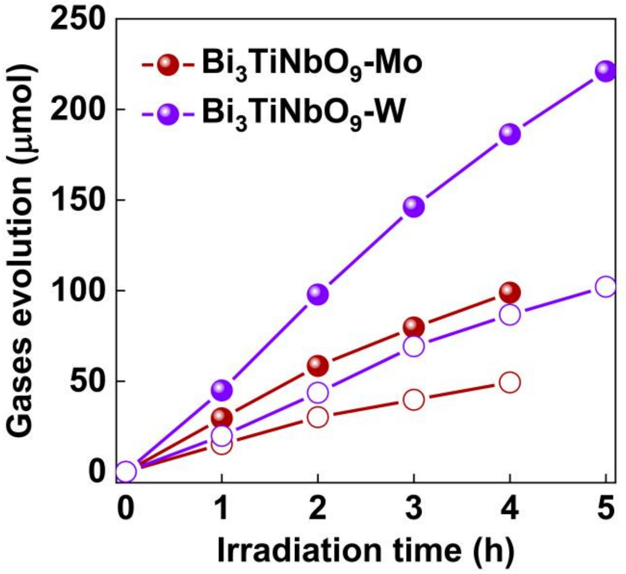

Supplementary Fig. 14 Comparison of photocatalytic overall water splitting activity of Bi_3TiNbO_9-W and Bi_3TiNbO_9-Mo.

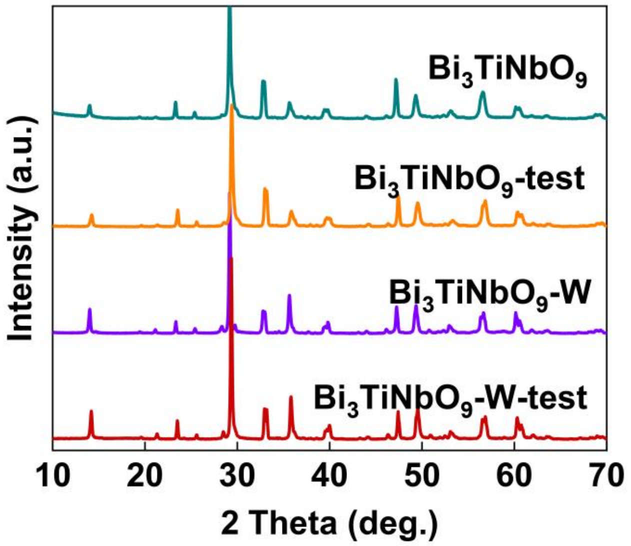

Supplementary Fig. 15 X-ray diffraction patterns of Bi_3TiNbO_9 and Bi_3TiNbO_9-W before and after photocatalytic overall water splitting reaction.

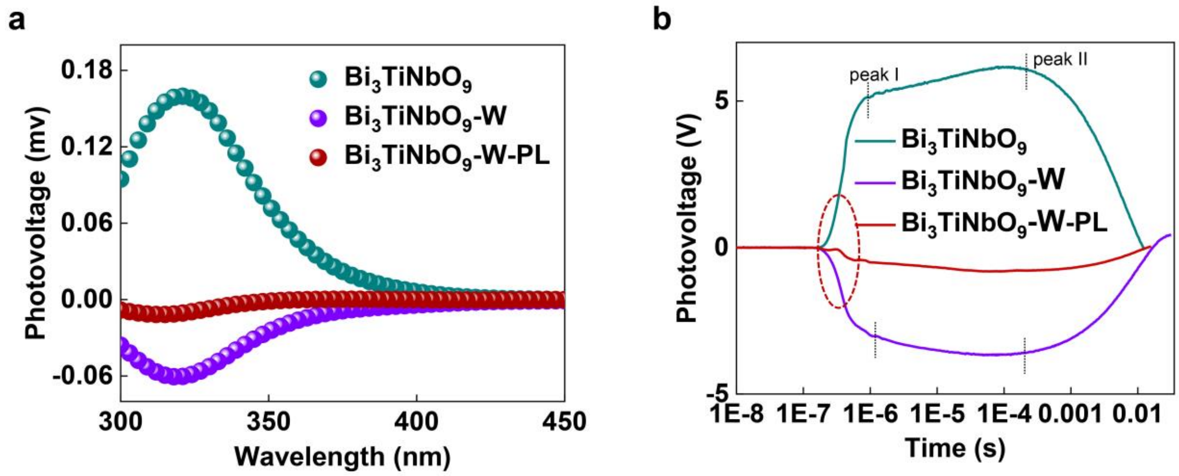

Supplementary Fig. 16 **a** Surface photovoltage (SPV) spectra and **b** transient photovoltage (TPV) response of multiple Bi_3TiNbO_9, Bi_3TiNbO_9-W, and Bi_3TiNbO_9-W-PL crystals.

Both Bi_3TiNbO_9 and Bi_3TiNbO_9-W exhibit n type semiconductor characteristics with upward surface band bending. Since the interlayer barrier and the surface space charge layer restrict the migration of photogenerated electrons to the surface (equivalent to the inherent built-in electric field, E_i, pointing from the bulk phase to the surface), Bi_3TiNbO_9 presents a positive SPV signal. When the direction of the additional built-in electric field (E) induced by gradient doping is opposite to E_i, the positive SPV signal intensity will be weakened, and even the direction of the SPV response will be reversed, for example, the W gradient doping in Bi_3TiNbO_9 presents a negative SPV signal. However, when the direction of the additional built-in electric field induced by gradient doping is the same as E_i, the positive SPV signal intensity will be enhanced[8, 9].

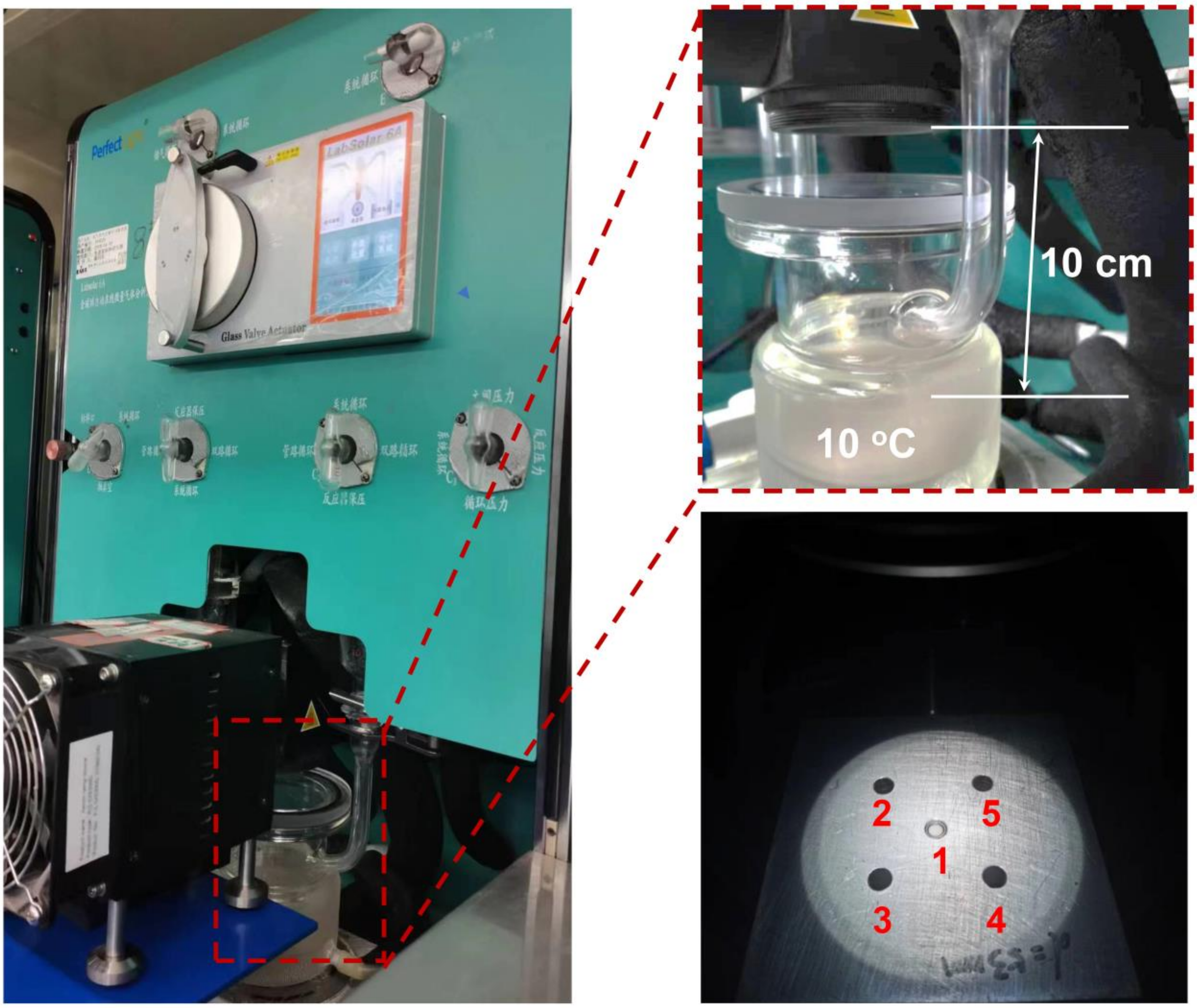

Supplementary Fig. 17 Equipment and operating status for photocatalytic water splitting.

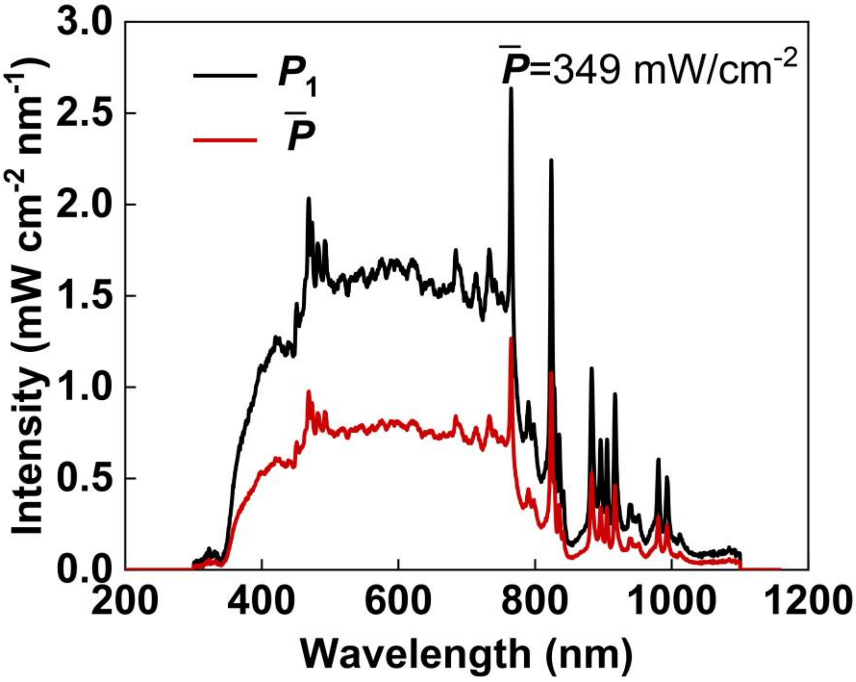

Supplementary Fig. 18 The spectrum of the light source used to measure photocatalytic water splitting.

Supplementary References

1. Huang, J. et al. Selective Exposure of Robust Perovskite Layer of Aurivillius-Type Compounds for Stable Photocatalytic Overall Water Splitting. *Adv. Sci.* **10,** 2302206 (2023).
2. Wan, G. et al. Photocatalytic Overall Water Splitting over $PbTiO_3$ Modulated by Oxygen Vacancy and Ferroelectric Polarization. *J. Am. Chem. Soc.* **144,** 20342-20350 (2022).
3. Liu, Y. et al. Bipolar charge collecting structure enables overall water splitting on ferroelectric photocatalysts. *Nat. Commun.* **13,** 4245 (2022).
4. Yin, X. et al. Realizing selective water splitting hydrogen/oxygen evolution on ferroelectric Bi_3TiNbO_9 nanosheets. *Nano Energy* **49,** 489-497 (2018).
5. Jiang, L., Ni, S., Liu, G., Xu, X. Photocatalytic hydrogen production over Aurivillius compound Bi_3TiNbO_9 and its modifications by Cr/Nb co-doping. *Appl. Catal. B* **217,** 342-352 (2017).
6. Bai, J., Chen, C., Zheng, J., Guo, C. Regulation of ferroelectric polarization and reduced graphene oxide (RGO) synergistically promoting photocatalytic performance of Bi_3TiNbO_9. *Mater. Today Phys.* **24,** 100691 (2022).
7. Park, Y., McDonald, K. J., Choi, K.-S. Progress in bismuth vanadate photoanodes for use in solar water oxidation. *Chem. Soc. Rev.* **42,** 2321-2337 (2013).
8. Liu, Y. et al. Internal-Field-Enhanced Charge Separation in a Single-Domain Ferroelectric $PbTiO_3$ Photocatalyst. *Adv. Mater.* **32,** 1906513 (2020).
9. Wang, W. C. et al. Surface photovoltage characterization of sol–gel derived $Bi_4Ti_3O_{12}$ ferroelectric thin film on F-doped SnO_2 conducting glass. *Chem. Phys. Lett.* **488,** 50-53 (2010).
